# Sensitivity of SARS-CoV-2 Life Cycle to IFN Effects and ACE2 Binding Unveiled with a Stochastic Model

**DOI:** 10.3390/v14020403

**Published:** 2022-02-15

**Authors:** Igor Sazonov, Dmitry Grebennikov, Andreas Meyerhans, Gennady Bocharov

**Affiliations:** 1 Faculty of Science and Engineering, Swansea University, Bay Campus, Fabian Way, Swansea SA1 8EN, UK; 2 Marchuk Institute of Numerical Mathematics, Russian Academy of Sciences (INM RAS), 119333 Moscow, Russia; 3 Moscow Center of Fundamental and Applied Mathematics at INM RAS, 119333 Moscow, Russia; 4 World-Class Research Center “Digital Biodesign and Personalized Healthcare”, Sechenov First Moscow State Medical University, 119991 Moscow, Russia; 5 Institució Catalana de Recerca i Estudis Avançats (ICREA), Pg. Lluis Companys 23, 08010 Barcelona, Spain; andreas.meyerhans@upf.edu; 6 Infection Biology Laboratory, Department of Experimental and Health Sciences (DCEXS), Universitat Pompeu Fabra, 08003 Barcelona, Spain; 7 Institute of Computer Science and Mathematical Modelling, Sechenov First Moscow State Medical University, 119991 Moscow, Russia

**Keywords:** SARS-Cov-2, type I interferon (IFN), the ACE2 receptor, virus dynamics, mathematical model, stochastic processes, Markov Chain Monte Carlo method, sensitivity analysis

## Abstract

Mathematical modelling of infection processes in cells is of fundamental interest. It helps to understand the SARS-CoV-2 dynamics in detail and can be useful to define the vulnerability steps targeted by antiviral treatments. We previously developed a deterministic mathematical model of the SARS-CoV-2 life cycle in a single cell. Despite answering many questions, it certainly cannot accurately account for the stochastic nature of an infection process caused by natural fluctuation in reaction kinetics and the small abundance of participating components in a single cell. In the present work, this deterministic model is transformed into a stochastic one based on a Markov Chain Monte Carlo (MCMC) method. This model is employed to compute statistical characteristics of the SARS-CoV-2 life cycle including the probability for a non-degenerate infection process. Varying parameters of the model enables us to unveil the inhibitory effects of IFN and the effects of the ACE2 binding affinity. The simulation results show that the type I IFN response has a very strong effect on inhibition of the total viral progeny whereas the effect of a 10-fold variation of the binding rate to ACE2 turns out to be negligible for the probability of infection and viral production.

## 1. Introduction

Severe acute respiratory syndrome coronavirus 2 (SARS-CoV-2) is the etiological agent of the coronavirus disease 2019 (COVID-19). The course and outcome of COVID-19 depend on multiple processes underlying the response of the host to the viral infection [1,2,3]. The resulting complexity calls for the application of mathematical modelling tools to describe, analyse, and predict the disease trajectories in relation to virus–host interaction parameters. This extension of the analytical tools needed to understand the pathogenesis of COVID-19 is highlighted by recent efforts to build up a computational resource linking available knowledge on the mechanisms of COVID-19 [4].

In patients infected with SARS-CoV-2, the race between the viral replication and the immune response determines the course of COVID-19 [3,5]. The immune determinants are key to explaining different disease progressions and outcomes [6] that are extensively analysed [2]. Heterogeneity in the spectrum of the infection dynamics could be potentially derived from viral variation [7]. Since the beginning of the SARS-CoV-2 pandemic, a number of new SARS-CoV-2 variants have emerged, such as the Alpha (B.1.1.7), Beta (B.1.351), Gamma (P.1), Delta (B.1.617.2), and more recently Omicron (B.1.1.529) [8,9,10,11]. However, it is not clear how the observed specific mutations increase the transmissibility and virulence of the virus [12].

To gain a mechanistic understanding of the relationship between a higher pathogenicity of SARS-CoV-2 variants and specific functions of the mutants, it is required to examine various sources of variability in viral infection/production bearing in mind that minor variations in replication rates could have a profound effect on viral loads [13]. The variations could be split into two categories. The first one is for those resulting from natural heterogeneity in virus production due to random effects and fluctuations in life cycle reactions. The second group is related to mutation-induced deterministic shifts in the kinetic parameters of SARS-CoV-2 replication, e.g., the receptor binding affinity, the susceptibility to type I interferon (IFN), and others.

The binding affinities between the Receptor Binding Domain (RBD) of the spike protein of SARS-CoV-2 variants and the ACE2 receptor have been recently characterised using a multidisciplinary approach combining all-atom steered molecular dynamics simulations and microscale thermophoresis [14]. It has been established that the Delta variant, upon the T478K mutation, requires the highest force for the RBD-ACE2 complex to be completely dissociated. Overall, the affinities of SARS-CoV-2 variants to ACE2 are higher than that of the wild-type (WT) with an increase factor ranging from 20–30% to 2.3-times. This links to the ability of the viral variants to infect a broader spectrum of target cells and results in a much higher infectivity, as shown in [15].

Upon infection with SARS-CoV-2, the type I IFN system is activated in the host cell [16]. IFN controls SARS-CoV-2 infection by inducing the expression of IFN-stimulated genes (ISGs) that restrict distinct steps of viral replication [17]. The detection of viral dsRNA is mediated by cytosolic innate sensors (RIG-I, MDA-5) and endosomal toll-like receptors TLRs (3,7,8) [18]. The type I IFN-mediated inhibition of SARS-CoV-2 growth in infected cells targets the translation initiation complex (PKR activation) and mRNA degradation (OAS activation) and induces an RNA editing enzyme (adenosine deaminase) [17].

We previously developed a deterministic mathematical model that describes the life cycle of SARS-CoV-2 in the form of ordinary differential equations (ODE) [19]. The model considers major replication stages including the binding of the virus to the ACE2 receptor, the translation of nonstructural proteins associated with the formation of a translation initiation complex, and RNA degradation. Hence, the model provides an appropriate tool that can be transformed into a stochastic form for examining the variability in the SARS-CoV-2 life cycle for the wild-type virus and its mutants. The latter are characterised by different affinities of their RBD affinity to the ACE receptor and their susceptibility to IFN. In this study, we develop a stochastic model of intracellular SARS-CoV-2 replication.

The stochasticity of the virus replication cycle can be accounted for by including Brownian motions into the deterministic model and by describing it via stochastic differential equations (SDE) [20,21,22]. However, the applicability of this approach is restricted by a daunting task of consistent estimation of the diffusion coefficients and the mode of noise-driven perturbations (additive or multiplicative) for all reaction stages in the model.

Another framework to model a stochastic infection dynamics is to employ a discrete or continuous Markov Chain (MC), which is implemented as a Dynamic Monte-Carlo method. Initially, it has been developed for chemical kinetics [23,24]. In this approach, the Markov chain and its parameters can be derived directly from the deterministic model. This provides an essential advantage over SDE-based modelling as the evaluation of parameters of the model is the most challenging problem. In our recent work [25], the MC-based stochastic modelling approach was successfully implemented to convert the mechanistic ODE model of HIV-1 life cycle into a stochastic Markov Chain Monte Carlo (MCMC) model [26]. The stochastic model enables us to quantify and explain the emergence of heterogeneities in the virus life cycle including the multiplicity of infection (MOI) and the variability in net viral progeny.

In this paper, we transform the deterministic model of the SARS-CoV-2 life cycle [19] into a stochastic model of the MCMC-type. We apply the stochastic model to examine the following statistical characteristics of a single-cell SARS-CoV-2 infection:

- The cell-to-cell variability in SARS-CoV-2 progeny production;
- The multiplicity of single cell infection;
- The probability of infection;
- The local sensitivity of the virus production to specific life-cycle steps;
- The impact of type I IFN; and
- The effect of the RBD-ACE2 binding affinity.

In Section 2, we formulate the stochastic model of the SARS-CoV-2 life cycle and describe its algorithmic implementation. Section 3 focuses on computational examination of the model behaviour for addressing various biologically relevant questions as listed above. The discussion of the results is presented in Section 4.

## 2. Methods

In this section, we introduce the deterministic ODE model of the SARS-CoV-2 life cycle developed in our previous work [19]. A formal notation is used for the time-dependent variables that are more suitable for the description and implementation of the stochastic MCMC model. The parameters and functional forms of the calibrated reaction kinetics are transformed into the propensity functions of the respective elementary reactions following the Gillespie approach [23]. The numerical implementation of the MCMC model is based on a hybrid stochastic-deterministic algorithm [25]. Finally, we introduce the approach to local sensitivity analysis of the stochastic model based on the computation of histogram differences for the ensembles of individually perturbed parameters.

### 2.1. Deterministic Equations of SARS-CoV-2 Single Cell Infection

**Figure 1:**
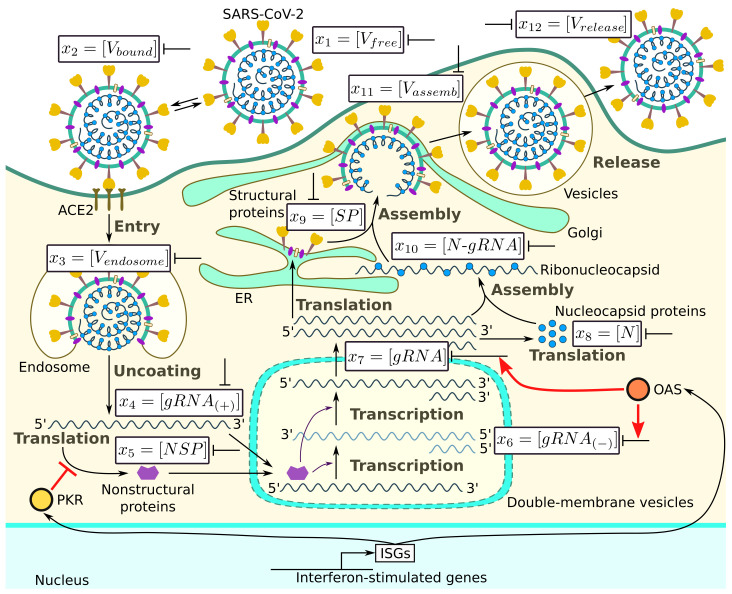
Biochemical scheme of the SARS-CoV-2 replication cycle. Targets of type I IFN-mediated inhibition of virus replication are marked.

The major steps of the intracellular life cycle of SARS-CoV-2 are schematically presented in Figure 1. In addition, we indicate the targets for type I IFN-mediated inhibition of virus replication.

According to this scheme, the infection process can be divided into several phases: (a) entry, (b) genome transcription and replication, (c) translation of structural and accessory proteins, and (d) assembly and release of virions. We consider all of these phases and present the corresponding equations following [19].

**Entry.** This phase is split into four stages:

(i) Binding of the receptor-binding domain (RBD) of the viral spike (S) protein to ACE2 receptor (Equation (1));
(ii) Priming of the virus S protein at the host cell surface by the transmembrane protease serine 2 (TMPRSS2), which leads to cleavage of the S proteins at the S1/S2 and S2 sites (Equation (2));
(iii) Fusion at the cellular or endosomal membrane (Equation (3)); and
(iv) Release and uncoating of viral genomic RNA (Equation (4)).

The population dynamics of the abundance of the respective molecular species is described by the following ordinary differential equations:

(1) $$\begin{array}{cc} {\dot{x}}_{1} & =-k_{\mathrm{bind}}x_{1}+k_{\mathrm{diss}}x_{2}-d_{V}x_{1} \end{array}$$

(2) x˙2=+kbindx1−kdissx2−kfusex2−dVx2

(3) x˙3=+kfusex2−kuncoatx3−dendosomex3

(4) x˙4=+kuncoatx3−dgRNAx4

where the dot over $x_{i}$ denotes the time derivative of $x_{i}$;

$x_{1}=\left[V_{\mathrm{free}}\right]$ is the number of free virions outside the cell membrane;
$x_{2}=\left[V_{\mathrm{bound}}\right]$ is the number of virions bound to ACE2 and activated by TMPRSS2;
$x_{3}=\left[V_{\mathrm{endosome}}\right]$ is the number of virions in endosomes; and
$x_{4}=\left[{\mathrm{gRNA}}_{\left(+\right)}\right]$ is the number of ss-positive sense genomic RNA.

**Genome transcription and replication.** This phase is split into three stages:

(v) The translation of the released genomic RNA into viral polyproteins (pp1a, pp1ab) which generate a number of non-structural proteins (nsp1-16), including nsp-12, which encodes the RNA-dependent RNA polymerase (RdRp) (Equation (5));
(vi) The RdRp-dependent transcription of a negative sense subgenomic and genomic RNAs (Equation (6)); and
(vii) The RdRp-dependent transcription of a positive sense subgenomic and genomic RNAs (Equation (7)).

The population dynamics of the abundance of the respective molecular species is described by the following ordinary differential equations:

(5) $$\begin{array}{cc} {\dot{x}}_{5} & =k_{\mathrm{transl}}f_{\mathrm{ORF}1}x_{4}-d_{\mathrm{NSP}}x_{5} \end{array}$$

(6) $$\begin{array}{cc} {\dot{x}}_{6} & =k_{{\mathrm{tr}}_{\left(-\right)}}\theta_{\mathrm{RdRp}}x_{4}-d_{{\mathrm{gRNA}}_{\left(-\right)}}x_{6} \end{array}$$

(7) $$\begin{array}{cc} {\dot{x}}_{7} & =k_{{\mathrm{tr}}_{\left(+\right)}}\theta_{\mathrm{RdRp}}x_{6}-k_{\mathrm{complex}}\theta_{\mathrm{complex}}x_{7}-d_{\mathrm{gRNA}}x_{7} \end{array}$$

where

$x_{5}=\left[\mathrm{NSP}\right]$ is the number of non-structural proteins;
$x_{6}=\left[{\mathrm{gRNA}}_{\left(-\right)}\right]$ is the number of negative sense genomic and subgenomic RNAs; and
$x_{7}=\left[\mathrm{gRNA}\right]$ is the number of positive sense genomic and subgenomic RNAs.

**Translation of structural and accessory proteins.** This phase is split into two major stages:

(viii) The translation of the structural nucleocapsid protein N from subgenomic RNAs by cytosolic ribosomes (Equation (8));
(ix) The translation of the structural proteins S, envelope E, and membrane M proteins characterised in the model by their total abundance [SP], which takes place in the endoplasmic reticulum (ER) (Equation (9)).

The population dynamics of the abundance of the respective molecular species is described by the following ordinary differential equations:

(8) $$\begin{array}{cc} {\dot{x}}_{8} & =k_{\mathrm{transl}}f_{N}x_{7}-k_{\mathrm{complex}}n_{N}\theta_{\mathrm{complex}}x_{7}-d_{N}x_{8} \end{array}$$

(9) $$\begin{array}{cc} {\dot{x}}_{9} & =k_{\mathrm{transl}}f_{\mathrm{SP}}x_{7}-k_{\mathrm{assemb}}n_{\mathrm{SP}}\theta_{\mathrm{assemb}}x_{10}-d_{\mathrm{SP}}x_{9} \end{array}$$

where

$x_{8}=\left[N\right]$ is the number of N proteins per virion and
$x_{9}=\left[\mathrm{SP}\right]$ is the total abundance of the structural proteins S, envelope E, and membrane M proteins.

**Assembly and release of virions.** This final phase is split into three major stages:

(x) The binding of N proteins and gRNA, resulting in nucleocapsid formation (viral RNA genome coated with N protein) (Equation (10));
(xi) The assembly of virions via encapsulating N-RNA complexes at the ER–Golgi compartment (Equation (11)); and
(xii) The release of the assembled new virions by the infected cell via exocytosis, budding, or cell death (Equation (12)).

The population dynamics of the abundance of the respective molecular species is described by the following ordinary differential equations:

(10) $$\begin{array}{cc} {\dot{x}}_{10} & =k_{\mathrm{complex}}\theta_{\mathrm{complex}}x_{7}-k_{\mathrm{assemb}}\theta_{\mathrm{assemb}}x_{10}-d_{N-\mathrm{gRNA}}x_{10} \end{array}$$

(11) $$\begin{array}{cc} {\dot{x}}_{11} & =k_{\mathrm{assemb}}\theta_{\mathrm{assemb}}x_{10}-k_{\mathrm{release}}x_{11}-d_{\mathrm{assemb}}x_{11} \end{array}$$

(12) $$\begin{array}{cc} {\dot{x}}_{12} & =k_{\mathrm{release}}x_{11}-d_{V}x_{12} \end{array}$$

where

$x_{10}=\left[N-\mathrm{gRNA}\right]$ is the number of ribonucleoprotein molecules;
$x_{11}=\left[V_{\mathrm{assembled}}\right]$ is the number of assembled virions; and
$x_{12}=\left[V_{\mathrm{released}}\right]$ is the number of released virions.

The following functions are present in Equations (6)–(11), which parameterise the saturation effects in the process kinetics of RNA transcription, nucleocapsid formation, and virion assembly, respectively:

(13) $$\theta_{\mathrm{RdRp}}=\frac{x_{5}}{x_{5}+K_{\mathrm{NSP}}},\;\theta_{\mathrm{complex}}=\frac{x_{8}}{x_{8}+K_{N}},\;\theta_{\mathrm{assemb}}=\frac{x_{9}}{x_{9}+K_{V_{\mathrm{rel}}}n_{\mathrm{SP}}}.$$

Thus, the SARS-CoV-2 replication dynamics is described by 12 ODEs (1)–(12), which we denote for convenience formally by variables $x_{1},\ldots ,x_{12}$. The system of equations is nonlinear because of nonlinear Michaelis–Menten-type functions (13).

### 2.2. Quantification of SARS-CoV-2 Replication Parameters

The calibration of the model, i.e., the estimation of parameters in the equations to reproduce the scale and kinetics of the SARS-CoV-2 life cycle was performed in our previous study [19].

The model parameters were calibrated using various data sources characterising (i) the biochemical properties of transcription and translation inherent to coronaviruses (CoV), (ii) the genomic organisation of SARS-CoV-2, (iii) the intracellular protein and RNA turnover, (iv) the in vitro growth data for recombinant (icSARS-CoV-Urbani, icSARS-CoV-GFP, and icSARS-CoVnLuc) and clinical strains of SARS-CoV2 (SARS-CoV-2 isolate WA1 and SARS-CoV-2 Australia/VIC01/2020).

The key sources of quantitative information on the respective processes include [26,27,28,29,30,31,32,33,34,35,36,37,38,39,40]). Note that all parameters of the model are characterised by some uncertainty (biologically plausible) ranges.

The basal set of the parameter estimates is presented in Table 1.

### 2.3. The Stochastic Model

A deterministic system described by ODEs can be translated into a stochastic description in the form of a Markov chain (MC): stochastic Dynamic Monte Carlo (DMC) approach. The stochastic framework considers the exact number of molecular species rather than a continuous approximation of their abundance. For low numbers of species with the interactions modeled following the chemical kinetics framework, an efficient algorithm for moving from a deterministic to probabilistic description of the trajectories has been proposed by Gillespie [23,24,41]. It has been shown that the solution of the MC describing the stochastic dynamics converges in probability to the solution of a related ODE system with proper scaling [42,43,44,45]. This limiting transition is called the fluid dynamics limit [42] or the mean field limit [46]. The theorem on a weak convergence of the MC process to the deterministic solution for specific models of viral infection dynamics has been proven in our earlier studies [45,47]. The list of elementary reactions, the corresponding transitions, and the propensities of the respective processes constituting the Markov chain stochastic model corresponding to the underlying ODEs (1)–(12) are presented in Table 2. The propensity function $a_{m}\left(x\right)$ is defined so that, for given current state x(t), the product $a_{m}\left(x\right)dt$ defines the probability that the *m*th reaction occurs in the infinitesimal time interval [t,t+dt] [24].

### 2.4. Stochastic Modelling Algorithm

To implement a MC numerically, a number of methods have been proposed, with the most popular being the Gillespie’s direct method [23,48,49]. In this method, the model state space vector $x=[x_{i},\ldots ,x_{N}]$ is specified by initial values for every component. Here, N=12 is the number of reaction components taking part in the replication process. In our case, the vector is initialised by setting all its components to zero except the first component, which is set to the initial number of free virions: $x_{1}=\left[V_{\mathrm{free}}\right]\left(0\right)$.

Then, the following steps are performed.

- At every interval between the reactions, two uniformly distributed random numbers $r_{1},r_{2}$ on (0,1) are generated. The first number gives the next timestep $\delta t=-(\ln r_{1})/A$, where $A=\sum_{m=1}^{M}a_{m}$; *M* is the number of reactions in the Markov chain; and $a_{m}$ is the propensity of the *m*th reaction: $a_{m}dt$ is the probability that the *m*th reaction occurs in time-interval dt. The second random number determines the next reaction index *p*: the smallest integer satisfying $A_{p}\geq Ar_{2}$, where $A_{p}=\sum_{m=1}^{p}a_{m}$. As we have to search among M=26 reactions, a binary search is employed to accelerate finding the reaction index *p* (see [49]). At the end of the step, the *p*th transition is performed, i.e., the state vector x is updated in accordance with Table 2.
- After updating the state vector, the propensities should be updated as well. Here, to accelerate the computation, the propensities are updated only for those reactions in which $a_{m}$ depends on the updated components in the given step. For this purpose, a special array is prepared in which propensities to be updated are indicated for given component $x_{n}$ and another similar array for every reaction *m*.
- The process is terminated as soon as the current time exceeds the maximal time $t_{\mathrm{final}}$ set in advance. To decrease the amount of stored information, the values of the state vector are stored at a uniform time-grid with the preset timestep Δt.

The algorithm is implemented in C++. To accelerate the computations, the arrays of pointers to functions are actively used to directly call functions of propensities that should be calculated for a given reaction without spending time on other reactions. The computations were run on Intel Xeon E3-1220 v5 CPU 3 GHz × 4. One realisation of the model with $\left[V_{\mathrm{free}}\right]\left(0\right)=10$ requires about 10 seconds of CPU time for $t_{\mathrm{final}}=48$ h. For every value of the initial number of free virions $\left[V_{\mathrm{free}}\right]\left(0\right)$, $10^{5}$ realisations are computed to obtain the statistically significant characteristics described in the next section. For $\left[V_{\mathrm{free}}\right]\left(0\right)=5$ and 10, as much as $10^{6}$ realisations are computed to obtain smoother histograms.

### 2.5. Local Stochastic Sensitivity Analysis

To perform a sensitivity analysis on the stochastic model, we followed the previously described approach for a local sensitivity analysis [25,50]. We obtained ensembles of the model outputs of interest (e.g., the model variable at a certain time, or some other functional of the model solution) with the baseline and perturbed model parameters. For each parameter $p_{i}$ perturbed by a small fixed percentage *s* of its baseline value, the sensitivity index can be defined by the histogram difference (sum of the absolute differences between the corresponding histogram bins):

(14) $$S_{i}=D\left(x_{0},x_{{\hat{p}}_{i}}\right),\;{\hat{p}}_{i}=p_{i}+\Delta p_{i}=p_{i}\left(1+s\right)$$

where $D(x_{0},x_{{\hat{p}}_{i}})$ is the histogram difference between the ensemble of model outputs $x_{0}$ obtained with baseline model parameters and the ensemble $x_{{\hat{p}}_{i}}$ obtained with parameter $p_{i}$ increased by a small value $\Delta p_{i}$. Employing this definition, the parameters can be ranked by their impact on the model output. The sensitivity indices can be compared to the so-called self-distance $D(x_{0},x_{0}^{\prime })$, i.e., the histogram difference between the ensembles obtained twice with the same baseline values of model parameters. The self-distance describes how well the histogram approximates the corresponding probability distribution function for a given number of stochastic model realisations in the ensemble. The sensitivity indices less than or close to the self-distance can be regarded as not having a strong effect [50]. Alternatively, the sensitivity indices can be defined as the histogram differences divided by the parameter variations $\Delta p_{i}$ and scaled on their baseline variables to make possible their ranking [25]. In this paper, we use definition (14) with one million realisations in each ensemble and s=0.01.

## 3. Results

### 3.1. Deterministic Versus Stochastic Dynamics of SARS-CoV-2 Replication

**Figure 2:**
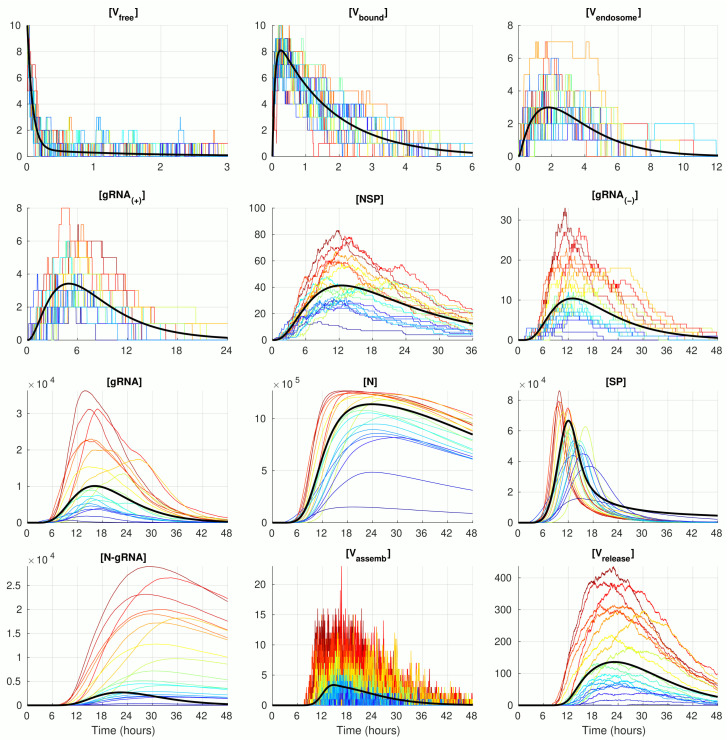
Examples of stochastic realisations for $\left[V_{\mathrm{free}}\right]\left(0\right)=10$. The black curves indicate the solution of the deterministic model.

The numerical solution of the deterministic ODE model of SARS-CoV-2 replication for the given number of virions infecting a single cell $\left[V_{\mathrm{free}}\right]\left(0\right)=10$, with the model parameter listed in Table 1 is shown in Figure 2 (black curve). The stochastic realisations of the MCMC model for $\left[V_{\mathrm{free}}\right]\left(0\right)=10$ are plotted by coloured lines (twenty arbitrarily taken realisations). The stochastic trajectories significantly deviate from the deterministic solution in both (i.e., up and down) directions. The lines with a more intensive red correspond to stochastic realisations with the highest peak of released virions $\left[V_{\mathrm{release}}\right]$. This enables tracing back the trajectories with higher (red and orange) and lower (green and blue) amplitudes of released SARS-CoV-2 through all components. By doing so, one can see that, beginning with the component [NSP], the red lines are strictly above the orange lines, which are in turn above the green and blue lines. Thus, fluctuations resulting in the number of released virions are determined at earlier stages of the virus life cycle process, i.e., in reactions involving $\left[{\mathrm{gRNA}}_{\left(+\right)}\right]$ and even $\left[V_{\mathrm{endosome}}\right]$ and $\left[V_{\mathrm{bound}}\right]$.

The trajectories for the components with high abundances such as [gRNA], [N], [SP], and [N-gRNA] look smoother because the stochastic effects of the reactions on their fluctuations are relatively small compared with the sizes of the respective molecular populations. In contrast, the trajectories for the number of $\left[V_{\mathrm{assemb}}\right]\left(t\right)$ display rather large and frequent fluctuations. This can be explained by the relatively large value of the parameter $k_{\mathrm{release}}$ (see Table 1), which results in a high probability of the assembled virions to be released from the infected cell, i.e., the newly formed virions stay in the intermediate assembled form for a short time.

### 3.2. Variability in Net Virus Production

**Figure 3:**
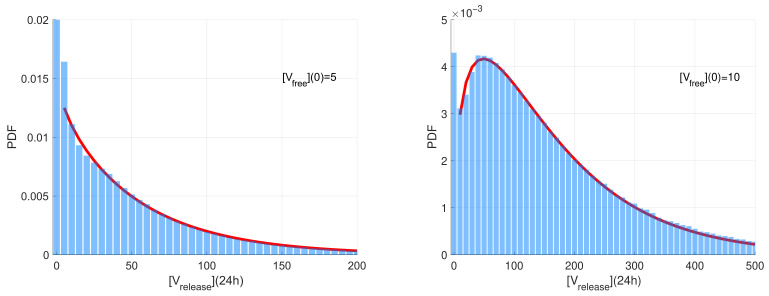
Normalised histograms for the released virions number for $\left[V_{\mathrm{free}}\right]\left(0\right)=5$ (**left**) and $\left[V_{\mathrm{free}}\right]\left(0\right)=10$ (**right**). The red line shows the approximation of the histogram by the Gamma distribution fitted to the histogram by the least squares method.

The computed stochastic realisations of the SARS-CoV-2 model were analysed to compute the statistical characteristics of the ensemble: the mean values, the medians, and quantiles. Two representative examples of the histograms of released virions at t=24 h post-infection generated for two different initial doses on infection $\left[V_{\mathrm{free}}\right]\left(0\right)=5$ (left) and $\left[V_{\mathrm{free}}\right]\left(0\right)=10$ are shown in Figure 3. The time t=24 h is selected because it is close to the peak time of production of SARS-CoV-2 virions. The histogram values are normalised with respect to the number of realisations and the range of the released number of virions considered in the histograms. Such normalisation enables the histogram to approximate the probability density function (PDF) describing the distribution.

The histograms have a noticeable peak at zero values for the number of released virions. This peak corresponds to extinct (degenerate) realisations in which no free virions are released. The complementary part of the histograms correspond to realisations characterised by a fully developed replication process resulting in the production of a significant number of free virions. This part of the histogram is rather smooth with an exponential tail. For $\left[V_{\mathrm{free}}\right]\left(0\right)=5$, this part is monotonically decaying and can be approximated by the exponential distribution. However, for $\left[V_{\mathrm{free}}\right]\left(0\right)=10$, it is non-monotone and shows a clear maximum. The corresponding part of the histogram can be approximated by the Gamma distribution $f\left(x\right)\propto x^{\alpha -1}e^{-\beta x}$ (note that the exponential distribution is its particular case) [51]. The results of the least-squares fitting of the Gamma distribution to the histograms (excluding the near-zero peak) are shown in Figure 3 as red curves.

### 3.3. Variability of the Individual Reaction Products

**Figure 4:**
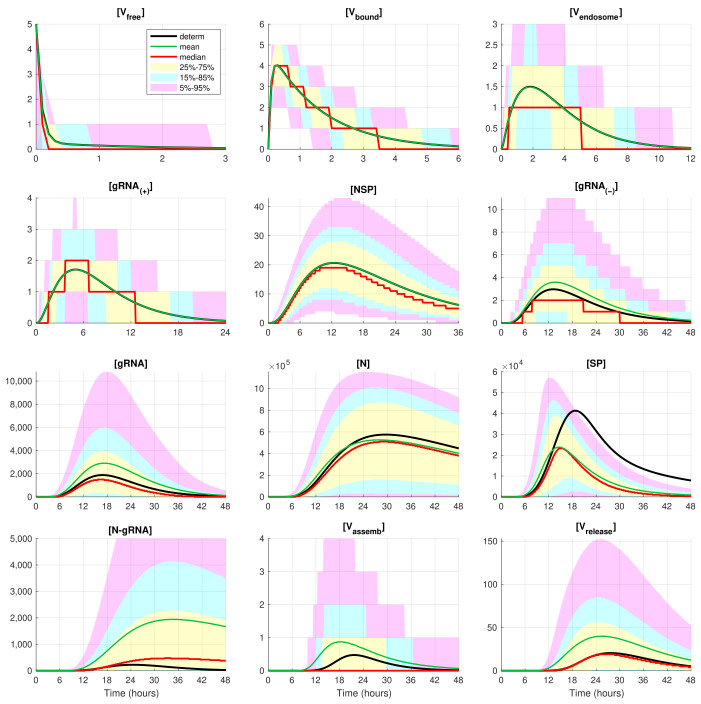
Evolution of the confidence intervals for all 12 species participating in the SARS-CoV-2 replication for $\left[V_{\mathrm{free}}\right]\left(0\right)=5$. The green, red, and black lines indicate the mean, median, and the deterministic solution, respectively.

**Figure 5:**
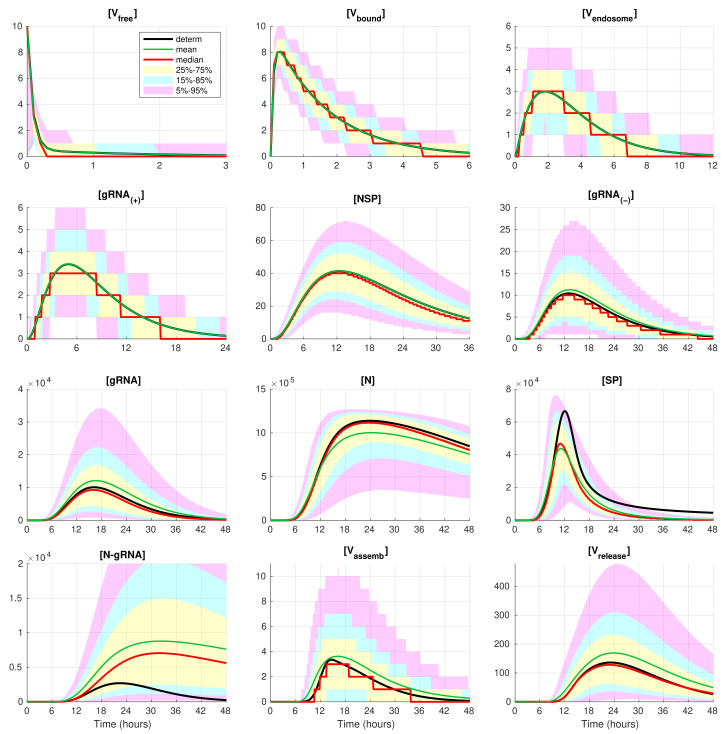
Evolution of the confidence intervals for all 12 components participating in the SARS-CoV-2 replication for $\left[V_{\mathrm{free}}\right]\left(0\right)=10$. The green, red, and black lines indicate the mean, median, and the deterministic solution, respectively.

The developed stochastic MCMC model allows us to systematically characterise the statistical properties of variability in the replication kinetics of SARS-CoV-2 emerging from the fluctuations in the underlying biochemical reactions and low numbers of reactants. To this end, an ensemble of $10^{5}$ realisations of the stochastic MCMC model is generated and analysed for the mean values, the medians, and uncertainty intervals in terms of various quantiles. The results are summarised in Figure 4 and Figure 5, corresponding to infection with 5 and 10 initial virions, respectively.

In these figures, the median values (50% quantile) are shown by the red lines, the green dashed lines indicate the mean values of the realisations, and the black lines indicate the solution to the deterministic model. The median curve can be treated as a trajectory of a so-called typical realisation [52]. As the histograms for components are mainly unimodal (opposite to the multimodal histograms of the stochastic HIV replication dynamics [25]), a comprehensive characterisation of the time uncertainty in the evolution of the SARS-CoV-2 components can be restricted to the quantiles of the sample distributions, which specify the related confidence intervals. In Figure 4 and Figure 5, the 25–75% confidence intervals (which include 50% of all realisations) are marked by yellow patches. The 15–85% confidence intervals are shown by the light-blue patches. They partly overlap with the 25–75% confidence intervals. The widest 5–95% confidence intervals (which include 90% of all realisations) are shown by the light-pink patches. The coloured patches in Figure 4 and Figure 5 provide quantitative details of the evolution of the histograms for all components during the development of the infection process.

Figure 4 and Figure 5 clearly show that the curves for the mean value trajectories exactly coincide with the deterministic curves for first five species of the SARS-CoV-2 replication steps considered in the model. This is because the first five ODEs (1)–(5) describing the kinetics of the respective species are linear. The first nonlinear equation is Equation (6) containing the nonlinear function $\theta_{\mathrm{RdRp}}$. Starting with $x_{6}=\left[{\mathrm{gRNA}}_{\left(-\right)}\right]$, there is a clear discrepancy between the sample mean values of the stochastic realisations and the deterministic curves. The most noticeable discrepancy is seen for [SP] and [N-gRNA]. Analysing the plots for components [N-gRNA], $\left[V_{\mathrm{assemb}}\right]$, and $\left[V_{\mathrm{release}}\right]$ in Figure 2, Figure 4 and Figure 5, one can conclude that some stochastic realisations have large deviations, significantly exceeding the deterministic and averaged trajectories. Their distributions are far from the Gaussian one.

The figures show that the deterministic solution is rather close to the sample median for $\left[V_{\mathrm{release}}\right]$. It is known that the median curves are useful for characterising stochastic processes as they represent the most probable trajectory. Note that, for $\left[V_{\mathrm{free}}\right]\left(0\right)=5$, the sample median curve is identical to zero for the component $\left[V_{\mathrm{assemb}}\right]$. This confirms our previous observation about a short availability time of the assembled virions inside an infected cell. The sample mean values for the number of released virions $\left[V_{\mathrm{release}}\right]$ are higher than the median values and the output deterministic solution, and the difference is larger for a smaller number of infection virions.

### 3.4. Probability of Productive Infection

Depending on the viral load and availability of permissive cells, the number of infecting viruses per cell (known as multiplicity of infection, MOI), denoted in our work as $\left[V_{\mathrm{free}}\right]\left(0\right)$, can vary substantially (see the discussion in [19]). We examine the effect of various MOI on the scale of virus replication and the probability of a non-degenerate infection. To this end, the numerical simulations have been performed for the initial number of virions $\left[V_{\mathrm{free}}\right]\left(0\right)=1,2,\ldots ,10$ over a representative time interval with $t_{\mathrm{final}}=48$ h (hours). This value of $t_{\mathrm{final}}$ exceeds the peak time $t_{\mathrm{peak}}∼24$ h at which the number of released virions attains its maximal value. Hence, the decreasing phase of the release process after the peak time is reproduced during the simulations as well.

**Figure 6:**
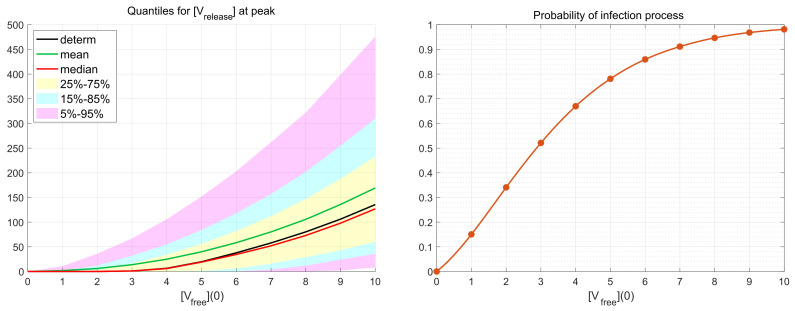
(**Left**) Dependence of confidence intervals, sample mean (green) and median (red) estimates, and the deterministic solution (black) on the initial number of free virions per cell $\left[V_{\mathrm{free}}\right]\left(0\right)$ at t=24 h. (**Right**) Probability for productive infection of the target cell in relation to the initial number of free virions per cell (MOI).

As it is mentioned above, realisations with zero number of newly produced virions, i.e., $\left[V_{\mathrm{release}}\right]\left(t\right)\equiv 0$, are called degenerate or extinct. The developed stochastic model enables the computation of the probability of such extinct cases depending on the MOI. The corresponding results are shown in Figure 6. New virions are produced in more than 50% of realisations beginning with $\left[V_{\mathrm{release}}\right]\left(0\right)=3$. This indicates the high infectious potential of SARS-CoV-2.

### 3.5. Efficiency of Life Cycle

The total number of new virions secreted by an infected cell during time *T* from the beginning of infection (thus, disregarding their degradation) is given by the formula

(15) $$\left[V_{\mathrm{new}}\right]\left(T\right)=\int_{0}^{T}k_{\mathrm{release}}\left[V_{\mathrm{assemb}}\right]\left(t\right)dt$$

obtained by integration of ODE (12) in which $d_{V}=0$ is set.

**Figure 7:**
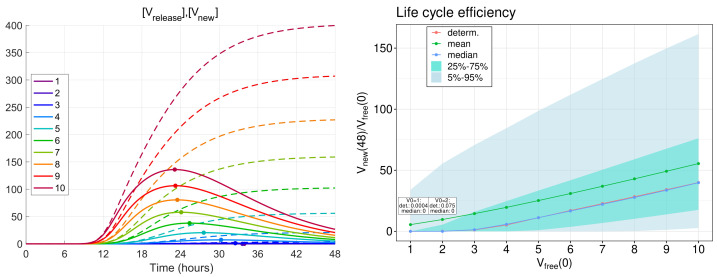
(**Left**) Kinetics of the total number of new virions secreted by an infected cell (dotted lines) for different MOIs (explained by the colour code) and the kinetics of virions release (solid lines) computed by the deterministic model [19]. (**Right**) The life cycle efficiency computed by the deterministic model (the red curve with circles). The mean, median, and the confidence intervals of the life cycle efficiency computed by the stochastic model (explained in the legend).

To examine the efficiency of a single life cycle of SARS-CoV-2, the kinetics of released virions were computed for the MOI, $\left[V_{\mathrm{free}}\right]\left(0\right)$, ranging from 1 to 10. The respective functions are shown in Figure 7 (left) by dashed lines. As one can see, all of the functions tend toward a finite limit, which gives the total viral progeny produced by the infected cell:

(16) $$\left[V_{\mathrm{total}}\right]=\lim_{T\to \infty }\left[V_{\mathrm{new}}\right]\left(T\right)=\int_{0}^{+\infty }k_{\mathrm{release}}\left[V_{\mathrm{assemb}}\right]\left(t\right)dt.$$

The analysis shows that $\left[V_{\mathrm{new}}\right]\left(T\right)$ for T=48 h approximately equals 99% of the total viral progeny $\left[V_{\mathrm{total}}\right]$. This means that computation within the time interval of 48 hours gives an appropriately accurate estimate for the total viral progeny: $\left[V_{\mathrm{new}}\right]\left(48\right)\approx \left[V_{\mathrm{total}}\right]$.

The life cycle efficiency can be characterised by the ratio of the total viral progeny to the MOI:

(17) $$\mathrm{Life cycle efficiency}=\frac{\left[V_{\mathrm{total}}\right]}{\left[V_{\mathrm{free}}\right]\left(0\right)}\approx \frac{\left[V_{\mathrm{new}}\right]\left(48\right)}{\left[V_{\mathrm{free}}\right]\left(0\right)}.$$

The dependence of the life cycle efficiency on MOI predicted by the deterministic model is presented in Figure 7 (right) by the blue line with circles. In the stochastic model, the life cycle efficiency varies from realisation to realisation; therefore, several statistical characteristics of this value are plotted in Figure 7 (right): the mean value—by the green line, the median—by the blue line, the 25–75%, and 5–95% confidence intervals—by the coloured patches.

One can see that the median values are close to the life cycle efficiency computed by the deterministic model, whereas the mean values are higher for all numbers of initial free virions. Note that the total viral progeny secreted by an ensemble of infected cells in tissue should be calculated by summing new virions produced by every cell. Then, just the mean value of the total viral progeny will fall on one cell. Therefore, the mean value of the life cycle efficiency looks to be a proper characteristic of the virion multiplication property of an infected cell.

The calculations show that the mean life cycle efficiency predicted by the stochastic model is noticeably higher than that obtained by the deterministic model, especially for lower MOI. For example, for five initial virions, the stochastic model gives a two times higher value for the life cycle efficiency than the same value obtained in the framework of the deterministic model. This difference indicates that the deterministic model can underestimate the contagiousness of SARS-CoV-2 and confirms the relevance of stochastic modelling of the virus life cycle.

### 3.6. Sensitivity Analysis of the Model Parameters

**Figure 8:**
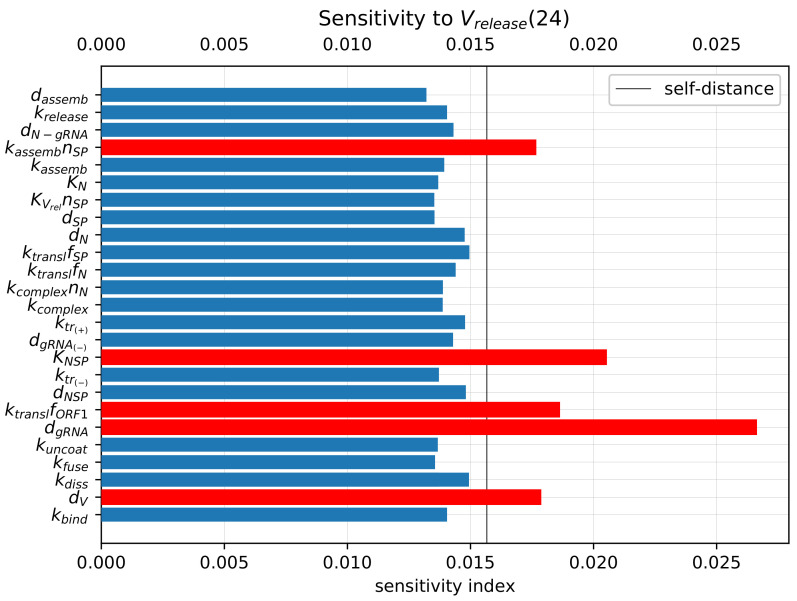
Local sensitivity indices for the number of released virions at 24 h computed with the stochastic model. The significant indices with greater values than the self-distance for the sets with baseline model parameters are marked by red.

To rank the parameters by their influence on the number of released virions, we apply the local sensitivity analysis (see Section 2.5). The sensitivity indices are based on the ensembles of variable $x_{12}=\left[V_{\mathrm{released}}\right]$ at time t=24 h for baseline values of model parameters and for parameters perturbed individually by 1%. The computed sensitivity indices as well as the self-distance for baseline set of parameters ($10^{6}$ realisations, 500 bins) are shown in Figure 8. The sensitivity indices for the model output $\left[V_{\mathrm{new}}\right]\left(24\right)$ reveal a similar pattern (data not shown).

The parameters with the largest influence on the model output (marked with the red colour bars in Figure 8) are similar to those obtained using the sensitivity analysis of the deterministic model [19]. They include the following (ranked by their impact):

- Degradation of the positive-sense vRNA in cytoplasm ($d_{\mathrm{gRNA}}$);
- Threshold number of non-structural proteins enhancing vRNA transcription ($K_{\mathrm{NSP}}$);
- Translation rate of non-structural proteins ($k_{\mathrm{transl}}f_{\mathrm{ORF}1}$);
- Degradation rate of extracellular virions ($d_{V}$); and
- Assembly rate of structural proteins ($k_{\mathrm{assembl}}n_{\mathrm{SP}}$).

Importantly, the parameters selected by sensitivity analysis correspond to the processes targeted by the type I interferon response. The effects of varying these parameters are studied in the next section.

### 3.7. Inhibitory Effect of Type I IFN

Type I Interferon responses are known to potently impair SARS-CoV-2 replication [13]. However, it has been reported that the induction of the type I IFN response and interferon-stimulated genes is moderate [16]. To characterise the sensitivity of the viral life cycle to the type I IFN response, we employed the stochastic model.

The IFN-mediated inhibition of SARS-CoV-2 growth in an infected cell targets the translation initiation complex (by protein kinase R (PKR) activation), and mRNA degradation (via 2${}^{\prime }$-5${}^{\prime }$-oligoadenylate synthetase (OAS) activation) and induces an RNA editing enzyme (adenosine deaminase) [17]. The corresponding effects in the parameterised mathematical model can be associated with variations in the following parameters:

(a) The rate constant of translation of released genomic RNA into viral polyproteins pp1a and pp1ab ($k_{\mathrm{transl}}f_{\mathrm{ORF}1}$);
(b) The degradation rate constant of RNA ($d_{\mathrm{gRNA}},\;d_{{\mathrm{gRNA}}_{\left(-\right)}}$); and
(c) = (a) + (b): simultaneous variation in both parameters.

**Figure 9:**
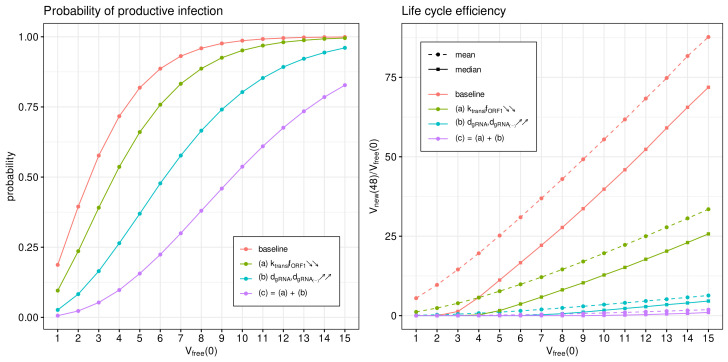
Type I IFN-mediated effects on the probability of non-degenerate infection (**left**) and the efficient reproduction number for MOI ranging from 1 to 15 (**right**). The ascending/descending arrows in the legend show the four-fold increase/decrease in the indicated parameters.

The parameters were increased by a factor of four with respect to the references values (see Table 1) to quantify the effect on the probability of a non-degenerate infection and the efficient reproduction number for a broad range of MOI, i.e., $\left[V_{\mathrm{free}}\right]\left(0\right)=1,...,15$. The results are summarised in Figure 9.

The probability of non-degenerate infection is shown in Figure 9 (left). Observe that the enhance IFN response significantly reduces the probability of the infection process developing. The effect is more pronounced for lower MOIs.

In Figure 9 (right), both the mean and median of the life cycle efficiency are plotted for the baseline and cases (a), (b) and (c) to characterise their partial and combined effects on viral progeny. One can see that the enhanced IFN response reduces new virus production by a factor exceeding 100.

### 3.8. Effect of Binding Affinity

The attachment of the spike protein of SARS-CoV-2 to the angiotensin-converting enzyme 2 receptor located on human cells is the first step of virus entry into host cells. It initiates the cascade of life cycle biochemical reactions [9,16]. It has been shown recently that the evolution of SARS-CoV-2 results in an emergence of viral variants of concern with the enhanced transmissibility and virulence [8]. Some mutations directly affect the affinity of the virus spike protein to the ACE2 receptor [12,15].

**Figure 10:**
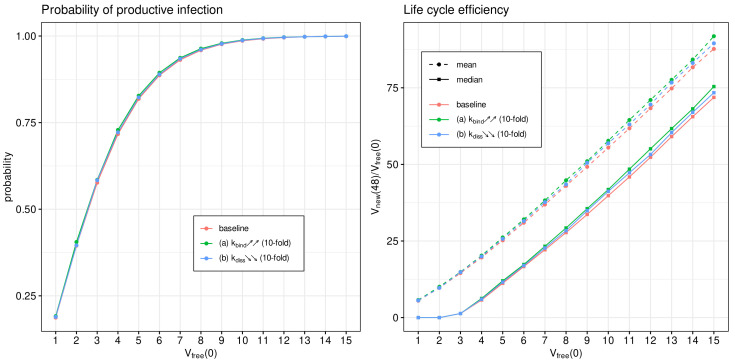
Effect of binding of SARS-CoV-2 to ACE2 on the probability of non-degenerate infection (**left**) and the efficient reproduction number (**right**) for MOI ranging from 1 to 15. The ascending/descending arrows in the legend show the 10-fold increase/decrease in the indicated parameters.

We use the developed model to examine the effect of binding affinity variation on the efficiency of the SARS-CoV-2 life cycle. To this end, the model parameter representing the rate constant of virion binding to the ACE2 receptor $k_{\mathrm{bind}}$ is varied from its basal value by 10 times to cover the range of observed increase or decrease in the binding affinities [14,53]. To quantify the binding rate effects on the probability of non-degenerate infections of host cells and the efficient reproduction number for a broad range of MOI ($\left[V_{\mathrm{free}}\right]\left(0\right)=1,\cdots ,15$), the parameter $k_{\mathrm{bind}}$ is increased (respectively, reduced) by a factor of 10 with respect to the reference value (see Table 1). The obtained results are summarised in Figure 10. Both the mean and median sample estimates are plotted to characterise their partial and combined effects on viral progeny.

One can see that the variation in the probability of infection and the net progeny of the life cycle is rather subtle. This feature might reflect the fact that an enhanced transmissibility of a certain SARS-CoV-2 mutants should be rather attributed to the ability of the variants to infect cells with the lower level of ACE2 receptors, which is consistent with the viewpoint in [15].

## 4. Discussion

We developed a stochastic model of SARS-CoV-2 replication in human cells. The model is formulated following the Dynamic Monte Carlo Markov Chain approach and utilises the calibrated parameters of our previously developed deterministic model [19]. Some predictions of the deterministic model might vary substantially for small numbers of molecular species participating in the virus life cycle, which is typical for a single cell infection. Numerical implementation of this model based on the Gillespie-type algorithm [23] enabled the calculation of all necessary statistical characteristics of the infection process variability. The probability for a non-degenerate infection process and the life cycle efficiency have been calculated for various MOI (i.e., the initial number of infecting viruses) and model parameters.

The simulation results suggest that the type I IFN response has a very strong effect on inhibition of the total viral progeny. This feature is consistent with the role of the type I IFN response to SARS-CoV-2 infection susceptibility [13]. Hence, our study supports the application of type I IFN as an early therapy [54]. Surprisingly, the effect of a 10-fold variation of the binding rate of SARS-CoV-2 to ACE2 turned out to be negligible for the probability of infection and viral production. This indicates that, in the analysis of the infectivity of the virus, it is necessary to go beyond a single cell infection and to consider the infection spreading in a population of host cells starting from low MOIs. It has been proposed recently that the greater infection efficiency of the SARS-CoV-2 Delta variant with its higher affinity for ACE-2 might be mainly due to the ability to infect cells with low numbers of ACE2 [14,15].

Recent data suggest that the Omicron variant is less effective at reducing the host cell interferon response [55]. Therefore, the intracellular IFN should have a stronger inhibitory effect on the virus replication for the Omicron mutant compared with the Delta variant, through targeting the translation initiation complex and mRNA degradation. Indeed, the sensitivity analysis of the stochastic model predicts a very strong suppressive impact of the type I IFN response on the probability of productive infection and the net viral progeny. These features should be relevant for understanding less severe disease courses observed in patients infected with the Omicron variant.

Our study provides a detailed model (overall, 12 life cycle intermediates) of the stochastic dynamics of SARS-CoV-2 replication in productively infected cells. This model can be regarded as a module for computational knowledge repository for studying the virus–host interaction mechanisms [4]. The virus replication stages considered in the study are inhibited by a type I IFN response of cells. However, we do not describe the induction and kinetics of the intracellular IFN response [18] and the factors used by SARS-CoV-2 (e.g., ORF3b, ORF6, and N proteins) to counteract the cellular innate immune response [16,56]. These will be the subject of future extensions of the model.

Understanding the variability in viral dynamics in infected host cells and its response to endogenous or exogenous perturbations of various nature is helpful for the development of effective antiviral treatments. Mathematical modelling of SARS-CoV-2 viral dynamics enables understanding the kinetic mechanisms and identifying potential therapeutic targets that can be useful for the development of efficient materials to suppress SARS-CoV-2 infection.

## Figures and Tables

**Table 1:** Reference parameter estimates of the deterministic model of SARS-CoV-2 replication.

$k_{\mathrm{bind}}$	= 12 h${}^{-1}$	$d_{V}$	=0.12 h${}^{-1}$	$K_{\mathrm{NSP}}$	= 100
$k_{\mathrm{diss}}$	=0.61 h${}^{-1}$	$d_{\mathrm{endosome}}$	=0.06 h${}^{-1}$	$K_{N}$	$=5\times 10^{6}$
$k_{\mathrm{fuse}}$	=0.5 h${}^{-1}$	$d_{\mathrm{gRNA}}$	=0.2 h${}^{1}$	$K_{V_{\mathrm{rel}}}$	= 1000
$k_{\mathrm{uncoat}}$	=0.5 h${}^{-1}$	$d_{\mathrm{NSP}}$	=0.069 h${}^{-1}$	$f_{\mathrm{ORF}1}$	= 1/21,000
$k_{{\mathrm{tr}}_{\left(-\right)}}$	= 3 h${}^{-1}$	$d_{{\mathrm{gRNA}}_{\left(-\right)}}$	=0.1 h${}^{-1}$	$f_{N}$	=1/1200
$k_{{\mathrm{tr}}_{\left(+\right)}}$	= 1000 h${}^{-1}$	$d_{N}$	=0.023 h${}^{-1}$	$f_{\mathrm{SP}}$	= 1/10,000
$k_{\mathrm{complex}}$	=0.4 h${}^{-1}$	$d_{\mathrm{SP}}$	=0.044 h${}^{-1}$		
$k_{\mathrm{transl}}$	$=4.536\times 10^{4}$ h${}^{-1}$	$d_{N-\mathrm{gRNA}}$	=0.2 h${}^{-1}$	$n_{N}$	= 456
$k_{\mathrm{assemb}}$	= 1 h${}^{-1}$	$d_{\mathrm{assemb}}$	=0.06 h${}^{-1}$	$n_{\mathrm{SP}}$	= 2000
$k_{\mathrm{release}}$	= 8 h${}^{-1}$				

**Table 2:** The Markov chain: the list of individual reactions, the corresponding state transitions, and the propensities of reactions.

*m*	Reaction (Transition)	Propensity, $a_{m}$	Equations
1	$\;x_{1}\to \;x_{1}-1,\;x_{2}\to x_{2}+1$	$k_{\mathrm{bind}}x_{1}$	(1), (2)
2	$\;x_{1}\to \;x_{1}+1,\;x_{2}\to x_{2}-1$	$k_{\mathrm{diss}}x_{2}$	(1), (2)
3	$\;x_{1}\to \;x_{1}-1$	$d_{V}x_{1}$	(1)
4	$\;x_{2}\to \;x_{2}-1,\;x_{3}\to x_{3}+1$	$k_{\mathrm{fuse}}x_{2}$	(2), (3)
5	$\;x_{2}\to \;x_{2}-1$	$d_{V}x_{2}$	(2)
6	$\;x_{3}\to \;x_{3}-1,\;x_{4}\to x_{4}+1$	$k_{\mathrm{uncoat}}x_{3}$	(3), (4)
7	$\;x_{3}\to \;x_{3}-1$	$d_{\mathrm{endosome}}x_{3}$	(3)
8	$\;x_{4}\to \;x_{4}-1$	$d_{\mathrm{gRNA}}x_{4}$	(4)
9	$\;x_{5}\to \;x_{5}+1$	$k_{\mathrm{transl}}f_{\mathrm{ORF}1}x_{4}$	(5)
10	$\;x_{5}\to \;x_{5}-1$	$d_{\mathrm{NSP}}x_{5}$	(5)
11	$\;x_{6}\to \;x_{6}+1$	$k_{{\mathrm{tr}}_{\left(-\right)}}\theta_{\mathrm{RdRp}}x_{4}$	(6)
12	$\;x_{6}\to \;x_{6}-1$	$d_{{\mathrm{gRNA}}_{\left(-\right)}}x_{6}$	(6)
13	$\;x_{7}\to \;x_{7}+1$	$k_{{\mathrm{tr}}_{\left(+\right)}}\theta_{\mathrm{RdRp}}x_{6}$	(7)
14	$\;x_{7}\to \;x_{7}-1,\;x_{10}\to x_{10}+1$	$k_{\mathrm{complex}}\theta_{\mathrm{complex}}x_{7}$	(7), (10)
15	$\;x_{8}\to \;x_{8}-1$	$n_{N}k_{\mathrm{complex}}\theta_{\mathrm{complex}}x_{7}$	(8)
16	$\;x_{7}\to \;x_{7}-1$	$d_{\mathrm{gRNA}}x_{7}$	(7)
17	$\;x_{8}\to \;x_{8}+1$	$k_{\mathrm{transl}}f_{N}x_{7}$	(8)
18	$\;x_{8}\to \;x_{8}-1$	$d_{N}x_{8}$	(8)
19	$\;x_{9}\to \;x_{9}+1$	$k_{\mathrm{transl}}f_{\mathrm{SP}}x_{7}$	(8)
20	$\;x_{9}\to \;x_{9}-1$	$n_{\mathrm{SP}}k_{\mathrm{assemb}}\theta_{\mathrm{assemb}}x_{10}$	(9)
21	$\;x_{9}\to \;x_{9}-1$	$d_{\mathrm{SP}}x_{9}$	(9)
22	$x_{10}\to x_{10}-1,\;x_{11}\to x_{11}+1$	$k_{\mathrm{assemb}}\theta_{\mathrm{assemb}}x_{10}$	(10), (11)
23	$x_{10}\to x_{10}-1$	$d_{N}x_{10}$	(10)
24	$x_{11}\to x_{11}-1$,$x_{12}\to x_{12}+1$	$k_{\mathrm{release}}x_{11}$	(11), (12)
25	$x_{11}\to x_{11}-1$	$d_{\mathrm{assemb}}x_{11}$	(11)
26	$x_{12}\to x_{12}-1$	$d_{V}x_{12}$	(12)

## Abbreviations

The following abbreviations are used in this manuscript:

RT	Reverse Transcription
ODE	ordinary differential equation
MC	Markov chain
MCMC	Markov chain Monte Carlo (method)
